# Characteristics and Outcomes of Patients with Acute Myocardial Infarction at Non-PCI Capable Hospitals in 2007 and in 2014

**DOI:** 10.1155/2015/359372

**Published:** 2015-10-04

**Authors:** Egle Kalinauskiene, Dalia Gerviene, Inga Sabeckyte, Albinas Naudziunas

**Affiliations:** Department of Internal Medicine, Medical Academy, Lithuanian University of Health Sciences, LT-47144 Kaunas, Lithuania

## Abstract

*Background*. There is little known about whether characteristics and outcomes of patients with acute myocardial infarction (AMI) have changed over the years in non-PCI capable hospitals in real-life. Our aim was to assess them between 2007 and 2014. *Methods*. It was a retrospective cohort study. Characteristics and in-hospital mortality (standardized in cases of different characteristics between the groups by original simple method) were assessed for all patients with non-ST elevation myocardial infarction (NSTEMI) and ST elevation myocardial infarction (STEMI) at two non-PCI capable hospitals: one in 2007 (*n* = 104) and another in 2014 (*n* = 58). *Results*. In 2014, females were older than in 2007 (80.18 ± 7.54 versus 76.15 ± 8.77, *p* = 0.011), males were younger (71.61 ± 11.22 versus 79.20 ± 7.63, *p* = 0.019), less had renal failure (RF) (19% versus 34.6%, *p* < 0.0001) and reinfarction (13.8% versus 35.6%, *p* < 0.0001), and the proportion of males (31% versus 43.3%, *p* = 0.001) and the proportion of NSTEMI (60.3 versus 69.2, *p* < 0.0001) decreased. In cases of STEMI there were no differences in patient characteristics. STEMI (18.8% versus 21.7%) and standardized mortalities by gender, RF, and reinfarction NSTEMI (19.47%, 15.34%, and 17.5%, resp., versus 17.1%) showed no differences between 2007 and 2014. *Conclusions*. There were some differences in patient characteristics but not in mortality for AMI at non-PCI capable hospitals between 2007 and 2014.

## 1. Introduction

There is little known about whether the clinical and demographical characteristics and in-hospital mortality of patients with acute myocardial infarction (AMI) have changed over the years in non-PCI capable hospitals (no team of interventional cardiologists) in real-life.

It was shown that life expectancy continues to increase [1, 2]. Consequently, the prevalence of age-related conditions, such as cardiovascular disease, is continuously increasing, and probably the age of patients with cardiovascular diseases is increasing, especially at non-PCI capable hospitals. Our previous study showed that patients treated conservatively in a non-PCI capable hospital and patients treated interventionally in a PCI capable hospital were significantly different: patients treated conservatively were much older, there were more women than men, and more often it was a non-ST elevation myocardial infarction (NSTEMI) than a ST elevation myocardial infarction (STEMI) [3]. Over the past several decades, the mortality rate for AMI has been decreasing with the development of reperfusion therapy and adjunctive pharmacological therapies [4]. However, most studies are done in the PCI capable hospitals and usually they are randomized clinical trials with their narrow inclusion criteria and wide exclusion criteria. The recent study showed discrepancies between trials and real-life: despite all current efforts, in-hospital mortality of patients with AMI was stagnating on a high level compared with data of randomized clinical trials [5].

Our aim was to assess the characteristics and outcomes of patients with acute myocardial infarction at non-PCI capable hospitals at two different periods: in 2007 and in 2014.

## 2. Material and Methods

It was a retrospective cohort study. Data of all patients hospitalized at the Kaunas Clinical Hospital (KCH) in 2007 and at the Republican Hospital of Kaunas (RHK) in 2014 with the diagnosis of an acute myocardial infarction (confirmed by the troponin test) were analysed. Both of these hospitals are non-PCI capable hospitals. The KCH was the main non-PCI capable hospital for conservative treatment of AMI in Kaunas city in 2007, and the RHK was the main non-PCI capable hospital for conservative treatment of AMI in Kaunas city in 2014. In both years patients received the same medications: heparin subcutaneously, dual antiplatelets therapy (aspirin and clopidogrel), B blockers, ACE inhibitors, and statins. No fibrinolytic therapy was administered, because in the same city there is a PCI capable hospital which has a skilled PCI laboratory with experienced interventional cardiologists on duty 24 h a day. A transfer for primary angioplasty or conservative treatment was chosen by the judgment of the attending cardiologist based on presenting characteristics and duration of symptoms. The enrolment of patients was consecutive and continued throughout the year. The study was approved by the Ethics Committee of the Lithuanian University of Health Sciences.

Patients of each hospital were divided into two groups according to electrocardiographic (ECG) changes on arrival to hospital: NSTEMI and STEMI [1]. Patients with conduction defects or electronic ventricular pacing were excluded. Patients were assessed by age, gender, comorbidities (diabetes mellitus (DM), renal failure (RF), or both of them (DM + RF)), reinfarction, echocardiographic left ventricular ejection fraction (LVEF), and the type of AMI (NSTEMI and STEMI). Their characteristics and in-hospital mortality were compared between the two hospitals, for example, between the two different periods of treatment at a non-PCI capable hospital in Kaunas city. Patients who arrived to RHK in 2014 also were divided into more detailed four subgroups according to ECG changes on arrival to the hospital: STEMI with positive T wave, STEMI with negative T wave, NSTEMI with positive T wave, and NSTEMI with negative T wave. Their age, gender, comorbidities, reinfarction, and LVEF also were compared and additional data as duration of chest pain before arriving to the hospital (<12 hours, 12–24 hours, and >24 hours) and high-sensitive troponin T level were compared between these subgroups.

In-hospital mortalities standardized by the frequency of some factors (which showed significant differences between the hospitals) were calculated by this original mathematical formula:

*x* = mortality of specific group, for example, RF in cases of NSTEMI in KCH*∗*frequency of RF in NSTEMI in RHK + *y∗*frequency of patients without RF with NSTEMI in RHK. *x*, in this example, the mortality of patients with NSTEMI in KCH, is standardized by RF (such mortality would be if the frequency of RF in KCH would be the same as in RHK in cases of NSTEMI); *y*, in this example, the mortality of patients without RF in cases of NSTEMI in KCH, is as follows:

(1) $$\begin{array}{c} y=\frac{\text{NSTEMI mortality in KCH}-\left(\text{NSTEMI mortality in cases of RF in KCH}*\text{frequency of RF in NSTEMI in KCH}\right)}{\text{frequency of patients without RF with NSTEMI in KCH}}. \end{array}$$

### 2.1. Statistical Analysis

Values were expressed as the mean ± standard deviation and as a percentage. Statistical significance was accepted when the probability value was *p* < 0.05. Differences in continuous variables between the two groups were assessed using unpaired Student's *t*-test and Mann-Whitney *U* test. Differences in continuous variables between more groups were assessed using One-Way ANOVA; comparisons of discrete variables were performed using Pearson's Chi-square test. Statistical analysis was performed using statistical package SPSS 21.0 and MS Excel.

## 3. Results

Patients hospitalized at the RHK in 2014 with the diagnosis of an AMI in comparison with the patients hospitalized at the KCH in 2007 were different in some characteristics: females were older, but males were younger, and less of the patients had RF and reinfarction (Table 1). And the proportion of males and the proportion of patients with NSTEMI decreased between them in comparison with the KCH in 2007. In 2007 in KCH NSTEMI was more frequent than STEMI, 69.2% versus 30.8%, *p* < 0.0001, while in 2014 in RHK the prevalence of NSTEMI was insignificant, 60.3% versus 39.7%, *p* > 0.05.

Progressive heart failure was the main cause of death in both hospitals: 12 cases (66.7%) at KCH in 2007 and 7 cases (63.6%) at RHK in 2014. Other causes were cardiogenic shock (4 cases) and arrhythmia (2 cases) at KCH in 2007, and cardiogenic shock (1 case), arrhythmia (1 case), cerebral stroke (1 case), pulmonic embolism (1 case) at RHK in 2014.

There were no significant differences in patient age, comorbidities, LVEF, and in-hospital mortality between STEMI and NSTEMI in each hospital, except that more males had NSTEMI than STEMI at KCH in 2007 (Table 2). However, there were no significant differences in male and female mortalities between STEMI and NSTEMI at each hospital.

In STEMI group there were no significant differences in patient characteristics and in-hospital mortalities at non-PCI capable hospitals between 2007 and 2014 (Table 3).

In NSTEMI group, significant differences were found between hospitals (years) in gender and frequency of patients with RF and reinfarction (Table 3). Therefore, sex-standardized in-hospital mortality and in-hospital mortalities standardized by the frequency of RF and reinfarction were calculated by our original mathematical formula.

Mortality of patients with NSTEMI in KCH standardized by RF (such mortality would be if the frequency of RF in KCH would be the same as in RHK in cases of NSTEMI) is as follows:

*x* = mortality of patients with RF in cases of NSTEMI in KCH*∗*frequency of RF in NSTEMI in RHK + *y∗*frequency of patients without RF with NSTEMI in RHK. *y* is the mortality of patients without RF in cases of NSTEMI in KCH. So, *x* = 20.7*∗*0.2 + *y∗*0.8,

(2) $$\begin{array}{c} y=\frac{16.7-\left(20.7*0.403\right)}{0.597}=14.0. \end{array}$$

So, mortality of 20% of patients is 20.7% and mortality of 80% of patients is 14.0%. Total mortality is *x* = 20.7*∗*0.2 + 14.0*∗*0.8 = 15.34. So, mortality of patients with NSTEMI in KCH in 2007 standardized by RF is 15.34%.

Mortality of patients with NSTEMI in KCH standardized by reinfarction is as follows:

(3) x14.8∗0.114+y∗0.886,y16.7−14.8∗0.3750.625=17.84,

so *x* = 17.5. So, mortality of patients with NSTEMI in KCH in 2007 standardized by reinfarction is 17.5%.

Sex-standardized mortality of patients with NSTEMI in KCH is as follows:

(4) x10.8∗0.286+y∗0.714,y16.7−10.8∗0.5140.486=22.94,

so *x* = 19.47. So, mortality of patients with NSTEMI in KCH in 2007 standardized by gender is 19.47%.

**Figure 1:**
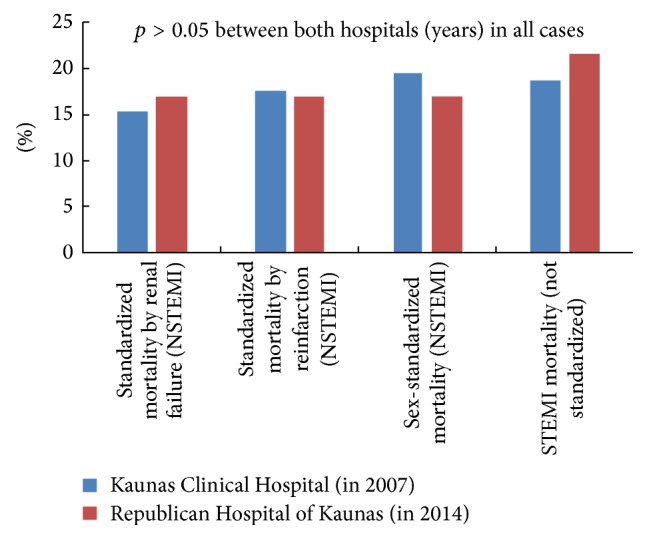
Sex-standardized and standardized by RF and reinfarction in-hospital mortality of patients with NSTEMI and not standardized in-hospital mortality of patients with STEMI at non-PCI capable hospitals between 2007 and 2014. In-hospital mortality of patients with STEMI was not standardized, because there were no differences in patient characteristics between both hospitals (years).

Sex-standardized and standardized by RF and reinfarction in-hospital mortality of patients with NSTEMI and not standardized in-hospital mortality of patients with STEMI are shown in Figure 1. In-hospital mortality of patients with STEMI was not standardized, because there were no significant differences in patient characteristics between both hospitals (years).

Comparison of more detailed ECG groups at RHK in 2014 did not show significant differences. There were no differences between all four subgroups of patients in age, gender, pain time, troponin level, LVEF, and in-hospital mortality (Table 4). However, some tendencies can be noted in this table. Troponin level tended to be greater in cases of STEMI and NSTEMI with negative T wave than in cases with positive T wave. In-hospital mortality tended to be lowest in cases of NSTEMI with negative T wave.

## 4. Discussion

Our study showed that there were some differences in patient characteristics at studied non-PCI capable hospitals between 2007 and 2014. In 2014, females were older, but males were younger, less of the patients had RF and reinfarction, and the proportion of males and patients with NSTEMI decreased. In 2007, NSTEMI was more frequent than STEMI, while in 2014 the prevalence of NSTEMI was insignificant. In cases of STEMI there were no differences in patient characteristics at non-PCI capable hospitals between 2007 and 2014. However, in cases of NSTEMI in 2014 less often than in 2007 patients were male and had RF and reinfarction.

Trends in AMI in 4 US states between 1992 and 2001 also showed an increase of patient age [6]. However, other studies showed that the proportion of patients with NSTEMI increased from 1990 to 2006 and from 2002 to 2011 [7, 8], in contrast to our study from 2007 to 2014. Also, McManus et al. reported that the incidence rates (per 100.000) of STEMI declined appreciably (121 to 77), whereas the incidence rates of NSTEMI increased slightly (126 to 132), between 1997 and 2005. Although in-hospital and 30-day case-fatality rates remained stable in both groups in that study, 1-year postdischarge death rates declined between 1997 and 2005 for patients with STEMI and NSTEMI [9]. But all these studies were done not at non-PCI capable hospitals in contrast to our study. More recent hospitalization-based analysis revealed a marked increase of NSTEMI among constant AMI frequency and showed discrepancies between trials and real-life: in-hospital mortality of patients with AMI was stagnating on a high level compared with data of randomized clinical trials [5]. Our study was a retrospective analysis of real-life data.

Characteristic of STEMI group was similar between the two hospitals. However, in NSTEMI group differences between hospitals in gender and frequency of patients with RF and reinfarction were found. Therefore, we calculated sex-standardized mortality and mortalities standardized by the frequency of RF and reinfarction by our simple method. Only in these three characteristics the difference was found, so we did not perform full standardization and did not calculate a risk-adjusted mortality by more complex models as it should be done in cases of comparison between many different hospitals with very different patients [10, 11]. Krumholz et al. showed that a simple 7-variable risk model performed as well as more complex models in comparing hospital outcomes for AMI. They concluded that although there is a continuing need to improve methods of risk adjustment, their results provide a basis for hospitals to develop a simple approach to compare outcomes [12]. The right choice of the comparison method is very important. One study, which compared four different methods across 83 hospitals in America, found that of 28 identified as the “worst” mortality hospitals by one company, 12 appeared in the “best” category when other methods were used [13]. However, our standardized mortalities showed no significant differences between in-hospital mortalities of neither patients with STEMI nor patients with NSTEMI in studied non-PCI capable hospitals between 2007 and 2014, as well as not standardized mortalities.

Analysis of more detailed ECG subgroups at a non-PCI capable hospital in 2014 showed no significant differences between all four subgroups of patients in age, gender, pain time, troponin level, LVEF, and in-hospital mortality. However, troponin level tended to be greater in cases of STEMI and NSTEMI with negative T wave than in cases with positive T wave in our study. It is possible that troponin tended to be greater for patients who arrived to hospital later with already inverted T wave, because troponin is increasing until the first 2–4 days of AMI. The patient-reported ischemic time (pain time in Table 4) showed no differences between the subgroups, but it is a subjective criterion. The recent study showed that terminal T wave inversion is a better predictor of outcomes in ST elevation MI than the patient-reported ischemic time and for patients undergoing urgent percutaneous coronary intervention it predicted worse outcomes [14]. However, in-hospital mortality tended to be lowest in cases of NSTEMI with negative T wave in our study performed at non-PCI capable hospitals. This subgroup may have included patients with not only NSTEMI, but also STEMI of late ECG stage with resolved ST segment elevation also, for example, who arrived to a hospital too late, and urgent percutaneous coronary intervention in such cases is not recommended, because it will not improve outcomes [15]. Therefore, the treatment at a non-PCI capable hospital is reasonable to such patients.

Limitations of our study are that two different hospitals were compared with the small cohort. However, the number of patients with AMI is decreasing in non-PCI capable hospitals. The main non-PCI capable hospital for conservative treatment of AMI in the same city was chosen in both years. This study was conducted in two similar non-PCI capable hospitals of the same city, but in two different periods.

## 5. Conclusions

In 2014 at a non-PCI capable hospital, females were older than in 2007, but males were younger, less of the patients had RF and reinfarction, and the proportion of males and the proportion of patients with NSTEMI decreased. In cases of STEMI there were no differences in patient characteristics at non-PCI capable hospitals between 2007 and 2014. In cases of NSTEMI in 2014 less often than in 2007 patients were male and had RF and reinfarction. There were no differences in sex-standardized in-hospital mortality and in-hospital mortalities standardized by the frequency of RF and reinfarction in cases of NSTEMI nor not standardized mortality in cases of STEMI between 2007 and 2014. We propose our used simple method of standardization for other comparisons of in-hospital mortalities in cases when groups are different only in few characteristics.

## Figures and Tables

**Table 1:** Characteristics and in-hospital mortality of patients with acute myocardial infarction at non-PCI capable hospitals in 2007 and in 2014.

Variable	KCH 2007 y. (*n* = 104)	RHK 2014 y. (*n* = 58)	*p*
Age (year)	77.47 ± 8.39	77.52 ± 9.6	0.564
Female age (year)	76.15 ± 8.77	80.18 ± 7.54	0.011
Male age (year)	79.20 ± 7.63	71.61 ± 11.22	0.019
Male *n* (%)	45 (43.3)	18 (31)	0.001
STEMI (%)	32 (30.8)	23 (39.7)	0.281
NSTEMI (%)	72 (69.2)	35 (60.3)	<0.0001
DM *n* (%)	7 (6.7)	5 (8.6)	0.774
RF *n* (%)	36 (34.6)	11 (19)	<0.0001
DM + RF *n* (%)	10 (9.6)	1 (1.7)	0.012
Reinfarction *n* (%)	37 (35.6)	8 (13.8)	<0.0001
LVEF (%)	37.91 ± 12.95	39.18 ± 11.68	0.598
In-hospital mortality *n* (%)	18 (17.3)	11 (19)	0.792
Mortality female *n* (%)	13 (22)	8 (20)	0.808
Mortality male *n* (%)	5 (11.1)	3 (16.7)	0.55

Data presented are mean value ± SD or number (percentage) of patients. Age and LVEF were compared using Mann-Whitney *U* test; other data were compared using Pearson's Chi-square test. KCH: Kaunas Clinical Hospital, RHK: Republican Hospital of Kaunas, STEMI: ST elevation myocardial infarction, NSTEMI: non-ST elevation myocardial infarction, DM: diabetes mellitus, RF: renal failure, DM + RM: diabetes and renal failure, and LVEF: left ventricular ejection fraction.

**Table 2:** Comparison of characteristics and in-hospital mortality between patients with STEMI and NSTEMI at non-PCI capable hospitals in 2007 and in 2014.

Variable	KCH 2007 y. (*n* = 104)			RHK 2014 y. (*n* = 58)		
	STEMI (*n* = 32)	NSTEMI (*n* = 72)	*p*	STEMI (*n* = 23)	NSTEMI (*n* = 35)	*p*
Age (years)	76.7 ± 8.85	77.8 ± 8.2	0.56	75.8 ± 8.86	78.6 ± 10.15	0.152
Female age (year)	77.4 ± 6.69	79.1 ± 7.16	0.371	78 ± 6.27	81.5 ± 8.04	0.059
Male age (year)	74.6 ± 13.9	76.59 ± 9.05	0.616	71.75 ± 11.32	71.5 ± 11.74	0.824
Female *n* (%)	24 (75)	35 (48.6)	0.193	15 (65.2)	25 (71.4)	0.154
Male *n* (%)	8 (25)	37 (51.4)	<0.0001	8 (34.8)	10 (28.6)	0.815
DM *n* (%)	2 (6.2)	5 (6.9)	0.896	2 (8.7)	3 (8.6)	0.987
RF *n* (%)	7 (21.9)	29 (40.3)	0.069	4 (17.4)	7 (20)	0.804
DM + RF *n* (%)	4 (12.5)	6 (8.3)	0.506	0	1 (2.9)	0.414
Reinfarction *n* (%)	10 (31.2)	27 (37.5)	0.539	4 (17.4)	4 (11.4)	0.519
LVEF (%)	34.1 ± 11.86	39.5 ± 13.14	0.054	36.6 ± 12.06	40.5 ± 11.81	0.243
In-hospital mortality *n* (%)	6 (18.8)	12 (16.7)	0.795	5 (21.7)	6 (17.1)	0.662
Female mortality *n* (%)	5 (20.8)	8 (22.9)	0.854	3 (20)	5 (20)	1.000
Male mortality *n* (%)	1 (12.5)	4 (10.8)	0.89	2 (25)	1 (10)	0.396

Data presented are mean value ± SD or number (percentage) of patients. Age was compared using Student's *t*-test in the Republican Hospital of Kaunas (RHK) and using Mann-Whitney *U* test in the Kaunas Clinical Hospital (KCH), also LVEF was compared using Mann-Whitney *U* test, other data were compared using Pearson's Chi-square test. STEMI: ST elevation myocardial infarction, NSTEMI: non-ST elevation myocardial infarction, DM: diabetes mellitus, RF: renal failure, DM + RM: diabetes and renal failure, and LVEF: left ventricular ejection fraction.

**Table 3:** Comparison of characteristics and in-hospital mortality of patients with STEMI and NSTEMI at non-PCI capable hospitals between 2007 and 2014.

Variable	STEMI			NSTEMI		
	KCH 2007 y. (*n* = 32)	RHK 2014 y. (*n* = 23)	*p*	KCH 2007 y. (*n* = 72)	RHK 2014 y. (*n* = 35)	*p*
Age (year)	76.7 ± 8.85	75.8 ± 8.86	0.71	77.8 ± 8.2	78.6 ± 10.15	0.678
Female age (year)	77.4 ± 6.69	78 ± 6.27	0.788	79.1 ± 7.16	81.5 ± 8.04	0.23
Male age (year)	74.6 ± 13.9	71.75 ± 11.32	0.657	76.59 ± 9.05	71.5 ± 11.74	0.146
Male *n* (%)	8 (25)	8 (34.8)	1.000	37 (51.4)	10 (28.6)	<0.0001
DM *n* (%)	2 (6.2)	2 (8.7)	0.73	5 (6.9)	3 (8.6)	0.746
RF *n* (%)	7 (21.9)	4 (17.4)	0.682	29 (40.3)	7 (20)	0.037
DM + RF *n* (%)	4 (12.5)	0	0.078	6 (8.3)	1 (2.9)	0.282
Reinfarction *n* (%)	10 (31.2)	4 (17.4)	0.244	27 (37.5)	4 (11.4)	0.005
LVEF (%)	34.1 ± 11.86	36.6 ± 12.06	0.454	39.5 ± 13.14	40.5 ± 11.81	0.72
In-hospital mortality *n* (%)	6 (18.8)	5 (21.7)	0.785	12 (16.7)	6 (17.1)	0.951
Mortality female *n* (%)	5 (20.8)	3 (20)	0.95	8 (22.9)	5 (20)	0.791
Mortality male *n* (%)	1 (12.5)	2 (25)	0.522	4 (10.8)	1 (10)	0.941

Data presented are mean value ± SD or number (percentage) of patients. Age in STEMI and NSTEMI group was compared using Student's *t*-test and LVEF using Mann-Whitney *U* test; other data were compared using Pearson's Chi-square test. KCH: Kaunas Clinical Hospital, RHK: Republican Hospital of Kaunas, STEMI: ST elevation myocardial infarction, NSTEMI: non-ST elevation myocardial infarction, DM: diabetes mellitus, RF: renal failure, DM + RM: diabetes and renal failure, and LVEF: left ventricular ejection fraction.

**Table 4:** Characteristics and in-hospital mortality of patients in electrocardiographic subgroups at the Republican Hospital of Kaunas (RHK) in 2014.

Variable	RHK 2014 y. (*n* = 58)				
	STEMI (*n* = 23)		NSTEMI (*n* = 35)		*p*
	Positive T wave (*n* = 10)	Negative T wave (*n* = 13)	Positive T wave (*n* = 11)	Negative T wave (*n* = 24)	
Age (years)	75.2 ± 10.29	76.31 ± 7.59	78.64 ± 6.99	78.63 ± 11.45	0.75
Female *n* (%)	5 (50)	10 (76.9)	10 (90.9)	15 (62.5)	0.171
Male *n* (%)	5 (50)	3 (23.1)	1 (9.1)	9 (37.5)	
Pain time <12 h *n* (%)	7 (70)	9 (69.2)	6 (54.5)	12 (50)	0.806
Pain time 12–24 h *n* (%)	1 (10)	0	1 (9.1)	2 (8.3)	
Pain time >24 h *n* (%)	2 (20)	4 (30.8)	4 (36.4)	10 (41.7)	
Troponin T hs (ng/L)	216 ± 49.07	1188.9 ± 584.4	829 ± 253.17	1134.5 ± 265.67	0.29
LVEF (%)	36 ± 10.59	37.08 ± 13.39	39.56 ± 12.9	41 ± 11.61	0.72
In-hospital mortality *n* (%)	2 (20)	3 (23.1)	3 (27.3)	3 (12.5)	0.73

Data presented are mean value ± SD or number (percentage) of patients. Age, troponin, and LVEF were compared using One-Way ANOVA; other data were compared using Pearson's Chi-square test. STEMI: ST elevation myocardial infarction, NSTEMI: non-ST elevation myocardial infarction, DM: diabetes mellitus, RF: renal failure, DM + RM: diabetes and renal failure, troponin T hs: troponin T high-sensitive, LVEF: left ventricular ejection fraction.
